# Identification of biomedical entities from multiple repositories using a specialized metadata schema and search-augmented large language models

**DOI:** 10.1186/s13104-026-07632-w

**Published:** 2026-01-12

**Authors:** Klaus Kaier, Felix Engel, Gita Benadi, Claudia Giuliani, Manuel Watter, Aref Kalantari, Karin Schuller, Claus-Werner Franzke, Markus Sperandio, Harald Binder

**Affiliations:** 1 Institute of Medical Biometry and Statistics, Medical Faculty and Medical Center, University of Freiburg, Freiburg, Germany; 2 Center for Integrative Biological Signaling Studies (CIBSS), University of Freiburg, Freiburg, Germany; 3 Helmholtz München, München, Germany; 4 Institute for Infection Prevention and Control, Medical Faculty and Medical Center, University of Freiburg, Freiburg, Germany; 5 Institute of Cardiovascular Physiology and Pathophysiology, Ludwig-Maximilians-Universität München, München, Germany

## Abstract

**Objective:**

Many biomedical articles reference multiple datasets across different public repositories, complicating accurate metadata capture and downstream re-use. Building on our prior grounded large language model (LLM) workflows for biomedical entity annotation, we extend the approach to identify and annotate all datasets referenced by a paper, even when distributed across repositories, by combining a specialized metadata schema with a three-step, search-augmented prompting strategy.

**Results:**

In the Transregional Collaborative Research Center PILOT (TRR 359 “Perinatal Development of Immune Cell Topology”), Gene Expression Omnibus (GEO) releases are common alongside additional repository deposits. The applied approach reliably detected datasets referenced in articles and produced schema-compliant annotations using information available on the repository landing pages. After validation through structured face-to-face interviews with the article’s senior author, Gemini 2.5 Pro achieved higher precision (97.1%) than GPT-4.1 (81.9%, *p* < 0.001) and Claude Sonnet 4 (88.6%, *p* < 0.001). Limiting the annotation to the information available in the repositories achieved higher precision than adding information from the article (919% vs. 88.3% across all LLMs, *p* = 0.004). These results indicate that simple repository-grounded extraction enables high quality, multi-dataset metadata annotation which has the potential to minimize the time and effort required for manual metadata annotation.

**Supplementary Information:**

The online version contains supplementary material available at 10.1186/s13104-026-07632-w.

## Introduction

Long-term research centers, such as Germany’s Collaborative Research Centers (CRCs), depend on structured data sharing and interoperable metadata to support FAIR-compliant reuse [1, 2]. In biomedicine, tagging entities like organisms, cell lines, or genes enhances data discoverability. Since manual annotation is slow and labor-intensive, automated methods such as named entity recognition are used (e.g., classifying “mouse” as an “organism”) [3]. The number and type of entities to extract depend on context and downstream analysis, varying with experimental focus (e.g., cell lines matter in in-vitro assays but not in population studies). Thus, entity selection must adapt to the task. For the CRC PILOT, which we consider as an exemplary setting, we developed a metadata schema with domain experts [4], previously used for manual annotation [5].

Many research articles now require to publish underlying datasets and more than one dataset are often distributed across multiple repositories (e.g., primary and secondary accessions, derived resources), which hinders comprehensive indexing, discovery, and integration. Prior to this work, we developed grounded large language model (LLM) workflows that combine a domain metadata schema with tool-use/validation to improve biomedical entity identification from research articles [6, 7].

Here, we address the specific challenge of automatically annotating multiple datasets per article. This fragmentation mirrors challenges seen in multi-institutional projects, where centralized, interoperable metadata platforms are proposed to unify discovery-level fields while linking discipline-specific standards across repositories [8]. The necessity for robust data harmonization extends to broader biomedical domains, as seen in complex workflows combining heterogeneous environmental and epidemiological datasets [9] or multi-repository metagenomic surveillance [10]. We introduce a three-step prompting approach that first enumerates dataset deposits per article, then anchors annotations to repository landing pages, and finally (optionally) enriches fields using the article when repository metadata are incomplete. The method is tailored to the PILOT context, where GEO submissions are frequent but additional deposits into other archives also occur. To assess the validity of this approach, a supervised double-checking process was conducted using face-to-face interviews. In summary, our work extends prior grounded-LLM approaches with three key contributions: (1) it explicitly handles the annotation of multiple datasets distributed across different repositories for a single publication; (2) it formalizes a repository-first grounding strategy, using landing pages as the primary source of truth to enhance precision and provenance; and (3) it validates this workflow within the context of a collaborative research center using a domain-specific metadata schema and expert review.

## Main text

### Methods

#### Prior workflow and setting

Our earlier studies evaluated grounded LLMs for biomedical entity identification using a schema-aware, multi-step generation and validation design, demonstrating high precision after expert verification [6, 7]. The present study builds on that foundation but shifts the primary evidence source from articles to repository landing pages to enhance faithfulness and reduce hallucinations, adding logic to support multiple datasets per manuscript. The evaluation was intentionally limited to six publications from the PILOT context, as the validation required supervised, researcher-author interviews. This design provided high-fidelity feedback but constrained sample size and external representativeness.

#### Metadata schema

We use a preliminary version of the PILOT metadata schema to standardize entities spanning, for example, *organism*, *timeline*, *cell line*, *tissue source*, *interventions*, *sample preparation/processing*, and *readout*, among others (controlled lists maintained with the project). The schema includes biomedical categories (e.g., *readout* and *sample processing*) and is designed for manual annotation by the responsible scientist [5]. The schema has a hierarchical structure with specific levels of data items triggering additional, more refined data items. For example, selecting ‘blood’ with the data item ‘tissue source’ would require an additional entry for data item ‘blood’ which offers a selection of blood components. As with this example, the names of data levels and data items often coincide.

#### Three-step, search-augmented prompting

We operationalize multi-dataset identification and annotation via a three-step prompting approach:

1. Identify datasets in public repositories. From the manuscript and its references, enumerate all dataset deposits and capture stable URLs/identifiers (e.g., GEO, SRA, ArrayExpress, PRIDE).
2. Extract biomedical entities from each repository page. Visit each dataset’s landing page and populate schema fields exclusively from the repository record (primary source of truth).
3. Enrich from the manuscript. When repository fields are incomplete, selectively consult the article to fill missing entries.

See Supplemental Fig. 1 for details on the prompting used in the three-step approach. For each manuscript, this three -step approach was repeated for three LLMs: GPT-4.1 (2025-04-14), Gemini 2.5 Pro (2025-06-17) and Claude Sonnet 4 (2025-05-22). All models were addressed via API calls using OpenRouter. To encourage diverse and exploratory outputs from the language models, we used a temperature of 1, top-p of 1, and top-k of 0 for each LLM. The search-augmentation was implemented using the OpenRouter system for web search via API. Each model received identical step prompts, schema definitions, and retrieval instructions. Finally, the results of step 2 and step 3 were evaluated separately:

Step 2: Repository-only content: biomedical entities were identified from the by repository pages only.

Step 3: Repository + article text: biomedical entities were identified from the by repository pages and additionally enriched using article information.

#### Evaluation

Primary endpoint was precision of schema-field annotations at the dataset level, where each individual annotation was the unit of analysis. Annotation precision was validated using face-to-face interviews between a research data management expert and the article’s senior author. Scientists reviewed each annotation against their datasets and, if needed, checked methods or results sections. Suspected inaccuracies were checked against the article’s methods or results, but annotations could also be rejected based on obvious errors or doubt without comprehensive cross-checking to maintain efficiency. To summarize the overall LLM accuracy in this exploratory setting, a descriptive meta-analysis of single proportions was conducted [11]. Because of small sample sizes and proportions near 1, the Freeman–Tukey double arcsine transformation was applied [12]. A random-effects model with REML accounted for between-study variability, and confidence intervals were calculated. P-values for between group comparisons were calculated using multilevel meta regression taking into account the multiple measurements of Steps or LLMs as random intercepts. Given the exploratory nature of the model comparisons, p-values were not adjusted for multiplicity. LLMs struggled with processing the conditional structure of the metadata schema (see Supplemental Fig. 1 for details on the schema), which was not conveyed in the prompts. Consequently, data levels were often formatted as data item names and vice versa. To handle this systematically, our evaluation protocol interpreted outputs as data levels where possible, counting these as correct suggestions. For example, if an LLM suggested a correct entity but failed to place it perfectly within the schema’s hierarchy, the entity itself was still marked as correct. This pragmatic approach was applied consistently across all models to ensure a fair comparison of their core entity recognition capabilities. Suggestions were also classified as correct if the required data level triggering a certain data item was not selected for a superordinated data item. These implied data entries, however, were not subsequently added to satisfy the schema’s inner logic and, consequently, did not contribute to the count of correct suggestions.

#### Reproducibility analysis

To assess the reproducibility of our findings, we re-implemented and re-ran the best-performing workflow (Step 2, using Gemini 2.5 Pro). This new implementation was developed within our internal data management system in preparation for larger-scale application. Minor technical modifications were required for this implementation, which are detailed in Supplemental Fig. 3. We then compared the set of biomedical entity annotations generated by the original run with those from the new implementation by calculating the Jaccard similarity index. The code for the implementation is publicly available at https://github.com/watterm/llm-metadata-annotation/tree/pilot-simplified.

### Results

Across six PILOT articles [13–18], the three-step pipeline successfully identified all datasets per paper and produced schema-compliant annotations using repository pages. All articles contained at least one data set with a link to a repository; the maximum number of linked data sets per article was 4. In detail, six datasets were available on the Gene Expression Omnibus (GEO) repository, 3 datasets were available in the European Nucleotide Archive (ENA), one data set was deposited in the Proteomics IDEmAS (PRIDE) database and one in the NCBI Sequence Read Archive (SRA). Last but not least, one set of processed data was published alongside analytical code on GitHub. See Supplemental Fig. 2 for details on all datasets used in the interviews.

**Fig. 1:**
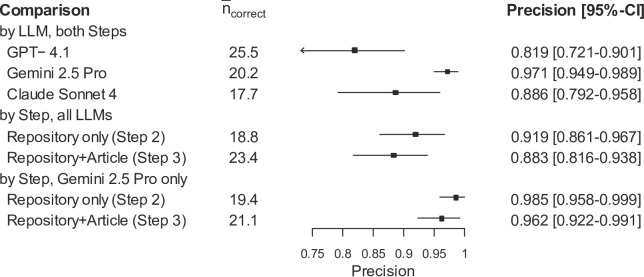
Identification of biomedical entities from multiple repositories using the PILOT metadata schema and search-augmented large language models.Step 2 refers to limiting the annotation to the information available in the repositories. Step 3 refers to the situation in which LLMs can use information from the article for metadata enrichment in addition to the repositories. $\:{\overline{\mathrm{n}}}_{\mathrm{c}\mathrm{o}\mathrm{r}\mathrm{r}\mathrm{e}\mathrm{c}\mathrm{t}}$ denotes the mean number of annotations classified as correct using face-to-face interviews between a research data management expert and the article’s senior author

The outcomes of step 2 are presented in Fig. 1. In this step, we restricted extraction to repository records only. Precision, defined as the share of correct annotations among all suggestions, ranged from 82 to 97%, indicating that the vast majority of predicted biomedical entities were considered correct in the face-to-face interviews. Gemini 2.5 Pro was associated with the highest precision 97.1% (95% CI 94.9%–98.9%), while Claude Sonnet 4 (*p* < 0.001) and GPT-4.1 (*p* < 0.001) performed worse. The mean number of correct entity annotations (expressed as $\:{\overline{n}}_{correct}$ in Fig. 1) was 20.2 for Gemini 2.5 Pro, 25.5 for GPT-4.1 and 17.7 for Claude Sonnet 4. The difference in the number of correct annotations versus precision highlights distinct model behaviors. For instance, GPT-4.1 generated a larger total volume of suggestions, resulting in more correct entities overall but also more errors, thus lowering its precision compared to the more conservative Gemini 2.5 Pro.

In addition, we found that limiting the annotation to the information available in the repositories achieved better results than adding information from the article. Across all LLMs, the precision was particularly higher in step 2 than in step 3 (91.9% vs. 88.3%, *p* = 0.004). At the same time, however the number of correct entity annotations increases when adding information from the article (see Fig. 1). When focusing of the results of the best-performing LLM (Gemini 2.5 Pro), the differences in the precision become less profound (98.5% vs. 96.2%, *p* = 0.105). Overall, the results suggest that the restriction to information from online repositories provide a good basis for LLM-supported metadata annotation.

To confirm the robustness of these findings, we conducted a reproducibility analysis of the best-performing approach (Step 2 with Gemini 2.5 Pro) using a separate implementation. The re-implementation was associated with a precision of 95.0%. The Jaccard similarity index between the annotations from the original run and the re-implementation was 0.62, indicating that while high precision was maintained, there was notable variability in the specific entities generated across runs (see Supplemental Fig. 3 for details).

### Discussion

This study demonstrates that search augmented, schema guided LLM pipelines can reliably discover and annotate multiple datasets per article when grounded in repository landing pages. Relative to article augmented extraction, repository only extraction improved precision—consistent with the intuition that repository records are curated, canonical source documents, whereas manuscript prose may include shorthand, future tense plans, or contextual statements that are easy for LLMs to overgeneralize. Viewed through a FAIR and AIready lens, repository-grounded extraction serves as a provenance spine to which model cards, prompts, training/evaluation artifacts, and compute context can be attached for robust reuse [19].

Our results extend earlier grounded LLM work on biomedical entity identification by (1) explicitly handling multi repository, multi dataset publications; (2) formalizing provenance aware field filling; and (3) providing a reusable prompt pack aligned to a biomedical metadata schema [6, 7]. While this study does not benchmark against other automated tools, it establishes the feasibility of this repository-grounded approach as a significant step towards semi-automating the currently manual and labor-intensive process of multi-dataset metadata curation. Such benchmarking will be incorporated in future work to contextualize performance relative to existing approaches. From a broader perspective, this repository-grounded approach aligns with community efforts to create more structured, machine-readable metadata, such as the BioSchemas and DataCite initiatives [20, 21]. By demonstrating that LLMs can effectively parse semi-structured repository pages into a custom schema, our work suggests a pathway for bridging existing repository metadata with more detailed, domain-specific schemas required by research consortia. The superior performance of Gemini 2.5 Pro may stem from its advanced multimodal capabilities and training on diverse web data, potentially making it more adept at interpreting the layout and content of scientific data repository pages compared to the other models tested.

## Limitations

The evaluation has several limitations. First, the sample size and scope were restricted to articles within the PILOT context, so generalizability to other domains and repositories remains to be tested at scale. Second, while expert review was used to create reference labels, residual subjectivity is still possible, which introduces a potential for investigator and respondent bias. This approach represents a trade-off between validation depth and scalability. Third, large language model outputs are inherently variable—non-deterministic and version-dependent—so exact replication requires pinning model versions and prompts. We did conduct a direct re-implementation of our best-performing model (Gemini 2.5 Pro, Step 2), which yielded consistent results (comparable precision, Jaccard similarity index = 0.62), suggesting practical robustness despite the model's stochastic nature. Fourth, the study prioritized precision over recall. This is due to the practical challenges of identifying all possible correct entities that were *not* predicted by the LLMs (i.e., false negatives). In natural language processing research, metrics such as recall and the F1 score are standard; however, in our open-ended, generative context, a finite and complete “ground truth” set of all relevant entities for a given dataset does not exist. The relevance of an entity is often subjective and context-dependent, making the total number of true positives and false negatives ill-defined. While, in principle, missing entities could be identified through exhaustive author interviews, this would require substantial additional effort. Nevertheless, by guiding the LLM with a predefined CRC metadata schema, the approach ensures that generated annotations are not only contextually appropriate but also relevant to the CRC framework. Last but not least, repository heterogeneity presents challenges, as field names and levels of detail vary across repositories, and some schema fields may be under-specified on landing pages.

Implementation of automated dataset annotation would have to respect the conditional inner logic of the metadata schema. Future research will have to address integration of this logic into the machine prompts (potentially leveraging techniques such as schema-guided decoding, tool-calling frameworks, or graph-based validation) in order to ensure compliant data population.

## Supplementary Information

Below is the link to the electronic supplementary material.

Supplementary Material 1.

Supplementary Material 2.

Supplementary Material 3.

## Data Availability

The datasets used and/or analysed during the current study are available from the corresponding author on reasonable request.
