# Supplementary material for: Identification of biomedical entities from multiple repositories using a specialized metadata schema and search-augmented large language models
Source: BMC Res Notes. 2026 Jan 12;19:43. doi: 10.1186/s13104-026-07632-w (PMC12837611; doi:10.1186/s13104-026-07632-w)
Supplement: Supplementary file 1 — Supplementary Material 1. [file 13104_2026_7632_MOESM1_ESM.pdf]

Supplemental Figure 1: Details on the prompt and schema

Step 1: Identification of datasets in public repositories
Analyze the uploaded manuscript to identify the datasets that were used in the article and published in public repositories. Try to identify the URLs to the datasets in the public repositories.
Do not provide additional information, but only list these datasets and the accompanying URLs.

Step 2: Identification of specific aspects within datasets
For each dataset identified in Step 1, use the URL and look at the website of the dataset. Use the information provided on the respective website to identify biomedical entities of the dataset. For structuring such entities, use the list of entities below. Maintain the formatting and organization provided below, and provide a comprehensive list for each subheading. Do not provide additional information or explanation, but only list the results according to the formatting and organization provided below.

- Organism
  - human
  - mouse
    - mouse line
      - beta-actin-dsRed
      - beta-actin-GFP
      - C57BL/6J (wildtype)
      - CAG-CreER
      - Ccr2-/-
      - Cd11c-Cre
      - Cd45.1
      - Clec4F-Cre
      - Csf2rb-/-
      - Csf2rb-flox
      - Cx3cr1-Cre
      - Cx3cr1-CreER
      - Cx3cr1-GFP
      - Cxcr4-CreER
      - DTA-flox
      - DTR-flox
      - gp91-/-
      - Hif1a-flox
      - Ifnar1-flox
      - iNos-/-
      - Irf8-/-
      - LysM-Cre
      - Mrc1-CreER
      - Ms4a3-Cre
      - MyD88-flox
      - Nav1.8-Cre
      - p47-/-
      - Pdgfra-CreER
      - Sftpc-CreER
      - Tgfbr2-flox
      - Tomato-flox
      - Villin-Cre
      - YFP-flox
      - C57BL/6J RccHsd (wildtype without nnt mutation)

        - Clec9a-cre x Rosa-tdTomato
        - Clec9a-cre x Rosa-YFP
        - Clec9a-cre x CD64-DTR
        - RORgt-eGFP
        - Cd11c-Cre x Stat1-flox
        - Clec9a-cre x Stat1-flox
        - Clec9a-cre x Irf8-flox
        - OT-I
        - OT-I x Nr4a1-eGFP
        - OT-II (Thy1.1)
        - Clec9a-cre x Irf4-flox-GFP
        - Clec9a-cre x EP2-KO x EP4-flox
    - pig
- timeline
    - embryonic / prenatal
    - perinatal – weaning
    - adult
- cell line
    - 3T3 cell
    - A2780
    - A549
    - AGS
    - AsPC-1
    - BEAS-2B
    - BHK-21
    - BT-474
    - BxPC-3
    - C2C12
    - C6
    - Caco-2 cell
    - Calu-3
    - Calu-6
    - Capan-1
    - Capan-2
    - CCRF-CEM
    - CHO cells
    - CHO-K1
    - COS-7
    - Daoy
    - DLD-1 cell
    - DU145
    - EA.hy926
    - F9
    - H1
    - H1299
    - H1975
    - H460
    - H9
    - HaCaT
    - HCT 116 cell
    - HEK293
    - HEK293T
    - HeLa

- HEP-2
- Hep3B
- HEPG2
- HL-60
- HL-60
- HOS
- HT-29
- Huh7
- HUVEC
- HUV-EC-C
- J774A.1 cell
- Jurkat
- Jurkat J6
- K562
- K562
- KG-1
- KG-1a
- L929
- LN229
- LNCaP clone FGC
- LoVo
- MCF10A
- MCF7 cell
- MDA-MB-231
- MDA-MB-453
- MDA-MB-468
- MDCK
- MEF (C57BL/6) cell
- MG-63
- MIA PaCa-2
- MKN28
- MKN-45
- MOLT-4
- MRC-5
- NB4
- NCI-H1299
- NCI-H1975
- NCI-H460
- NIH-3T3
- NT2-D1
- NTERA-2 cl.D1
- OVCAR-3
- OVCAR-4
- P19
- PANC-1
- PC-3
- PLC/PRF/5
- Raji
- Ramos
- RAW 264.7 cell
- RWPE-1
- Saos-2
- SH-SY5Y

  - SK-BR-3
  - SK-N-SH
  - SK-OV-3
  - SupT1
  - SW480
  - SW620
  - T47D
  - T98G
  - THP-1
  - U251
  - U2OS
  - U87MG
  - U937
  - U937
  - Vero
  - WI-38
- tissue source
  - Blood
    - Blood
      - plasma
      - serum
      - whole blood
      - Blood cells
  - Bone marrow
  - Embryonal tissue
  - Heart
  - Intestine
  - Liver
  - Lung
  - Lymph node
  - Nerve
  - Skin
    - Skin
      - Dermis
      - Epidermis
      - Fascia
  - Spleen
  - Thymus
  - Vascular system
    - Vascular system
      - Abdominal aorta
      - Aortic arch
      - Aortic root
      - Inferior vena cava
      - Superior vena cava
  - Adipose tissue
  - CNS
    - CNS
      - Brain
      - Spinal cord
      - Dura
      - Leptomeninges
  - Small Intestine

    - Colon
    - Kidney
- Interventions
    - Genetic modification
        - Genes of interest
            - TGFB1
            - MRC1
            - MYD88
            - GP91
            - P47
            - HIF1A
            - NOS2
    - Pharmacological treatment
        - Pharmacological/dietary interventions
            - High fat diet
            - Tamoxifen induction
            - DSS treatment
            - Csf1R inhibitor treatment
            - Diphteria toxin treatment
            - Antibiotics
    - Infection
        - Infection
            - Bacille Calmette Guérin (BCG)
            - Guillain-Barre syndrome (GBS)
            - Staphylococcus aureus
            - Cytomegalovirus
            - Mycobacterium marinum
            - Mycobacterium avium
            - Legionella pneumophila
    - Injection
        - Injection
            - Poly I:C
            - Maternal inflammation
            - CpG
            - R848
    - Housing condition
        - Housing condition
            - Individual housing
            - Germ-free
- Sample preparation
    - Cultured cells
        - Cell type
            - Adipocyte
            - Dendritic cell
            - Embryonic cardiomyocytes
            - Endothelial cell
            - Epithelial cell
            - Fibroblast
            - hiPSC-CM
            - hiPSC-FB
            - Immune cell
            - Leukocytes

        - Lymphocytes
        - Macrophage
        - Monocytes
        - Natural killer cells
        - Neuronal cell
        - Neurones
        - Neutrophils
        - Oocyte
        - Pericytes
        - Platelet
        - Smooth muscle cells
        - T cells
        - B cell
    - Isolated cells
        - Cell type
            - Adipocyte
            - Dendritic cell
            - Embryonic cardiomyocytes
            - Endothelial cell
            - Epithelial cell
            - Fibroblast
            - hiPSC-CM
            - hiPSC-FB
            - Immune cell
            - Leukocytes
            - Lymphocytes
            - Macrophage
            - Monocytes
            - Natural killer cells
            - Neuronal cell
            - Neurones
            - Neutrophils
            - Oocyte
            - Pericytes
            - Platelet
            - Smooth muscle cells
            - T cells
            - B cell
    - Tissue chunk
    - Tissue section (thin)
    - Tissue slice
    - Whole organ
- Sample processing
    - Cleared fixed tissue
    - Formaldehyde fixed and parafin embedded (FFPE)
    - Formaldehyde-fixation
    - High pressure frozen
    - None (Physiological solution)
    - OCT embedded and frozen
- Readout
    - Biomechanics
        - Biomechanics
            - Cell stretching

    - Nanoindentation
    - Sarcomer Length
    - Single cell stretching
    - Tissue stretching
    - Tissue stretching Myodish
- Clinical Imaging
  - Clinical Imaging
    - Computed tomography scan
    - Echocardiography
    - Magnetic resonance imaging
    - Positron emission tomography
- Computational modelling
  - Computational modelling
    - Quantitative Trait Locus (QTL)
    - Differential Gene Expression (DGE)
    - Cell type analysis
- Electron microscopy
- Flow cytometry / FACS
  - Cytometry
    - Fluorescence
    - Confocal
- Light microscopy
  - Light microscopy
    - Confocal
    - Multiphoton
    - Slidescanner
    - Widefield
    - Fluorescence
- Cellular/molecular biology
  - Cellular/Molecular biology
  - Cytokine immune profiling
  - Cell subset composition
  - Cell function & cell states
  - Epigenomics
  - Transcriptome
  - Inflammation
  - Metabolomics
- Optical mapping
- Optoacoustics
- Sequencing
  - Sequencing
    - ATAC sequencing
    - DNA methylation (WGBS)
    - RNA sequencing
    - Single Cell chromatin accessibility
    - Single cell RNA seq
    - Whole exome sequencing
    - Whole genome sequencing
    - CITE-seq

Structure your results in unformatted plan text with only the entity categories with a simple "-" up-front. Here is an example of the category "Organism" and the result "mouse":

-Organism
Mouse

Complete this step for every dataset entirely before proceeding to the next step.

Step 3: Use information from the manuscript to enrich the information regarding the datasets
Use the results of Steps 2. In addition, reanalyze the manuscript and try to add missing information for each dataset using the list of entities from Step 2. Complete this step for every dataset entirely before proceeding to the next step. Do not provide additional information, but only list the results according to the formatting and organization provided before.
