# Supplementary material for: Identification of biomedical entities from multiple repositories using a specialized metadata schema and search-augmented large language models
Source: BMC Res Notes. 2026 Jan 12;19:43. doi: 10.1186/s13104-026-07632-w (PMC12837611; doi:10.1186/s13104-026-07632-w)
Supplement: Supplementary file 2 — Supplementary Material 2. [file 13104_2026_7632_MOESM2_ESM.pdf]

Supplemental Figure 2: Results of all meta analyses

Study	Dataset	LLM	Step	n_{total}	$n_{correct}$	Proportion [95%−CI]
Forde 2023	GSE174676	GPT− 4.1	Step 2	67	65	0.970 [0.896, 0.996]
Forde 2023	GSE174676	GPT− 4.1	Step 3	87	75	0.862 [0.771, 0.927]
Forde 2023	GSE174676	Gemini 2.5 Pro	Step 2	48	47	0.979 [0.889, 0.999]
Forde 2023	GSE174676	Gemini 2.5 Pro	Step 3	82	71	0.866 [0.773, 0.931]
Forde 2023	GSE174676	Claude Sonnet 4	Step 2	22	22	1.000 [0.846, 1.000]
Forde 2023	GSE174676	Claude Sonnet 4	Step 3	40	38	0.950 [0.831, 0.994]
Baasch 2021	GSE159389	GPT− 4.1	Step 2	36	21	0.583 [0.408, 0.745]
Baasch 2021	GSE159389	GPT− 4.1	Step 3	44	25	0.568 [0.410, 0.717]
Baasch 2021	GSE159389	Gemini 2.5 Pro	Step 2	24	24	1.000 [0.858, 1.000]
Baasch 2021	GSE159389	Gemini 2.5 Pro	Step 3	23	23	1.000 [0.852, 1.000]
Baasch 2021	GSE159389	Claude Sonnet 4	Step 2	19	18	0.947 [0.740, 0.999]
Baasch 2021	GSE159389	Claude Sonnet 4	Step 3	34	23	0.676 [0.495, 0.826]
Baasch 2021	PXD015923	GPT− 4.1	Step 2	27	16	0.593 [0.388, 0.776]
Baasch 2021	PXD015923	GPT− 4.1	Step 3	19	16	0.842 [0.604, 0.966]
Baasch 2021	PXD015923	Gemini 2.5 Pro	Step 2	11	11	1.000 [0.715, 1.000]
Baasch 2021	PXD015923	Gemini 2.5 Pro	Step 3	16	14	0.875 [0.617, 0.984]
Baasch 2021	PXD015923	Claude Sonnet 4	Step 2	11	11	1.000 [0.715, 1.000]
Baasch 2021	PXD015923	Claude Sonnet 4	Step 3	16	15	0.938 [0.698, 0.998]
Torow 2023	GSE188714	GPT− 4.1	Step 2	47	19	0.404 [0.264, 0.557]
Torow 2023	GSE188714	GPT− 4.1	Step 3	67	22	0.328 [0.218, 0.454]
Torow 2023	GSE188714	Gemini 2.5 Pro	Step 2	17	16	0.941 [0.713, 0.999]
Torow 2023	GSE188714	Gemini 2.5 Pro	Step 3	19	17	0.895 [0.669, 0.987]
Torow 2023	GSE188714	Claude Sonnet 4	Step 2	37	19	0.514 [0.344, 0.681]
Torow 2023	GSE188714	Claude Sonnet 4	Step 3	37	17	0.459 [0.295, 0.631]
Torow 2023	PRJNA794356	GPT− 4.1	Step 2	22	14	0.636 [0.407, 0.828]
Torow 2023	PRJNA794356	GPT− 4.1	Step 3	40	18	0.450 [0.293, 0.615]
Torow 2023	PRJNA794356	Gemini 2.5 Pro	Step 2	6	6	1.000 [0.541, 1.000]
Torow 2023	PRJNA794356	Gemini 2.5 Pro	Step 3	7	7	1.000 [0.590, 1.000]
Torow 2023	PRJNA794356	Claude Sonnet 4	Step 2	21	10	0.476 [0.257, 0.702]
Torow 2023	PRJNA794356	Claude Sonnet 4	Step 3	28	9	0.321 [0.159, 0.524]
Rohwedder 2023	GSE222156	GPT− 4.1	Step 2	33	32	0.970 [0.842, 0.999]
Rohwedder 2023	GSE222156	GPT− 4.1	Step 3	38	36	0.947 [0.823, 0.994]
Rohwedder 2023	GSE222156	Gemini 2.5 Pro	Step 2	11	11	1.000 [0.715, 1.000]
Rohwedder 2023	GSE222156	Gemini 2.5 Pro	Step 3	11	11	1.000 [0.715, 1.000]
Rohwedder 2023	GSE222156	Claude Sonnet 4	Step 2	10	10	1.000 [0.692, 1.000]
Rohwedder 2023	GSE222156	Claude Sonnet 4	Step 3	33	29	0.879 [0.718, 0.966]
Mansoori Moghadam 2024	GSE263475	GPT− 4.1	Step 2	28	14	0.500 [0.306, 0.694]
Mansoori Moghadam 2024	GSE263475	GPT− 4.1	Step 3	33	18	0.545 [0.364, 0.719]
Mansoori Moghadam 2024	GSE263475	Gemini 2.5 Pro	Step 2	18	17	0.944 [0.727, 0.999]
Mansoori Moghadam 2024	GSE263475	Gemini 2.5 Pro	Step 3	15	15	1.000 [0.782, 1.000]
Mansoori Moghadam 2024	GSE263475	Claude Sonnet 4	Step 2	21	15	0.714 [0.478, 0.887]
Mansoori Moghadam 2024	GSE263475	Claude Sonnet 4	Step 3	33	18	0.545 [0.364, 0.719]
Mansoori Moghadam 2024	PRJEB70607	GPT− 4.1	Step 2	16	15	0.938 [0.698, 0.998]
Mansoori Moghadam 2024	PRJEB70607	GPT− 4.1	Step 3	19	18	0.947 [0.740, 0.999]
Mansoori Moghadam 2024	PRJEB70607	Gemini 2.5 Pro	Step 2	12	12	1.000 [0.735, 1.000]
Mansoori Moghadam 2024	PRJEB70607	Gemini 2.5 Pro	Step 3	11	11	1.000 [0.715, 1.000]
Mansoori Moghadam 2024	PRJEB70607	Claude Sonnet 4	Step 2	12	12	1.000 [0.735, 1.000]
Mansoori Moghadam 2024	PRJEB70607	Claude Sonnet 4	Step 3	15	15	1.000 [0.782, 1.000]
Mansoori Moghadam 2024	PRJEB70609	GPT− 4.1	Step 2	13	13	1.000 [0.753, 1.000]
Mansoori Moghadam 2024	PRJEB70609	GPT− 4.1	Step 3	16	16	1.000 [0.794, 1.000]
Mansoori Moghadam 2024	PRJEB70609	Gemini 2.5 Pro	Step 2	14	14	1.000 [0.768, 1.000]
Mansoori Moghadam 2024	PRJEB70609	Gemini 2.5 Pro	Step 3	10	10	1.000 [0.692, 1.000]
Mansoori Moghadam 2024	PRJEB70609	Claude Sonnet 4	Step 2	12	12	1.000 [0.735, 1.000]
Mansoori Moghadam 2024	PRJEB70609	Claude Sonnet 4	Step 3	13	13	1.000 [0.753, 1.000]
Mansoori Moghadam 2024	PRJEB70610	GPT− 4.1	Step 2	13	13	1.000 [0.753, 1.000]
Mansoori Moghadam 2024	PRJEB70610	GPT− 4.1	Step 3	15	15	1.000 [0.782, 1.000]
Mansoori Moghadam 2024	PRJEB70610	Gemini 2.5 Pro	Step 2	13	13	1.000 [0.753, 1.000]
Mansoori Moghadam 2024	PRJEB70610	Gemini 2.5 Pro	Step 3	12	12	1.000 [0.735, 1.000]
Mansoori Moghadam 2024	PRJEB70610	Claude Sonnet 4	Step 2	12	12	1.000 [0.735, 1.000]
Mansoori Moghadam 2024	PRJEB70610	Claude Sonnet 4	Step 3	14	14	1.000 [0.768, 1.000]
Halgouet 2024	GSE222454	GPT− 4.1	Step 2	28	26	0.929 [0.765, 0.991]
Halgouet 2024	GSE222454	GPT− 4.1	Step 3	42	39	0.929 [0.805, 0.985]
Halgouet 2024	GSE222454	Gemini 2.5 Pro	Step 2	33	31	0.939 [0.798, 0.993]
Halgouet 2024	GSE222454	Gemini 2.5 Pro	Step 3	33	31	0.939 [0.798, 0.993]
Halgouet 2024	GSE222454	Claude Sonnet 4	Step 2	28	28	1.000 [0.877, 1.000]
Halgouet 2024	GSE222454	Claude Sonnet 4	Step 3	31	30	0.968 [0.833, 0.999]
Halgouet 2024	github_gdTcells	GPT− 4.1	Step 2	28	26	0.929 [0.765, 0.991]
Halgouet 2024	github_gdTcells	GPT− 4.1	Step 3	42	39	0.929 [0.805, 0.985]
Halgouet 2024	github_gdTcells	Gemini 2.5 Pro	Step 2	33	31	0.939 [0.798, 0.993]
Halgouet 2024	github_gdTcells	Gemini 2.5 Pro	Step 3	33	31	0.939 [0.798, 0.993]
Halgouet 2024	github_gdTcells	Claude Sonnet 4	Step 2	3	2	0.667 [0.094, 0.992]
Halgouet 2024	github_gdTcells	Claude Sonnet 4	Step 3	34	32	0.941 [0.803, 0.993]

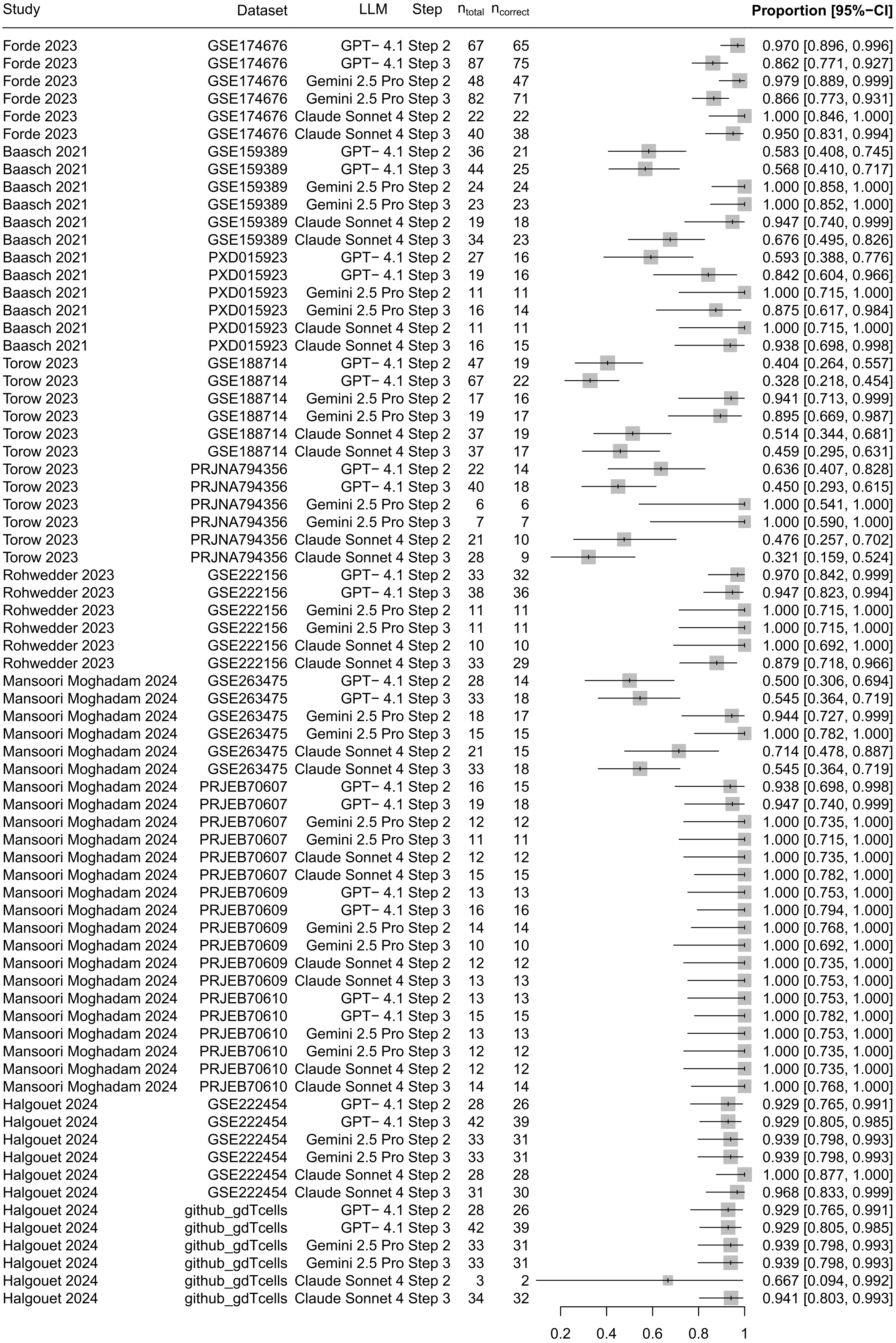

Note: github_gdTcells refers to a datset published at https://github.com/sagar161286/multimodal_gdTcells/
