# Supplementary material for: Identification of biomedical entities from multiple repositories using a specialized metadata schema and search-augmented large language models
Source: BMC Res Notes. 2026 Jan 12;19:43. doi: 10.1186/s13104-026-07632-w (PMC12837611; doi:10.1186/s13104-026-07632-w)
Supplement: Supplementary file 3 — Supplementary Material 3. [file 13104_2026_7632_MOESM3_ESM.pdf]

Supplemental Figure 3: Results of the rerun of the best-performing approach (Step 2 with Gemini 2.5 Pro) using a new implementation developed for fitting the data with our internal data management system

To assess the reproducibility of our findings, we re-implemented and re-ran the best-performing workflow (Step 2, using Gemini 2.5 Pro). This new implementation was developed within our internal data management system in preparation for larger-scale application. Minor technical modifications were required for this implementation. In detail, we used Mistral OCR to convert the article PDF to Markdown. The LLM was instructed to provide dataset identifiers and URLs as JSON via structured output in the Step 1 response. This allowed us to generically automate retrieval of the data repository web page content via Playwright, which is subsequently filtered and cleaned to reduce irrelevant HTML content. Besides this information, the instructions and output for Steps 2 and 3 are identical to the original setup.

We then compared the set of biomedical entity annotations generated by the original run (Figure 3a) with those from the new implementation (Figure 3b) and by calculating the Jaccard similarity index (Figure 3c). The code for the implementation is publicly available at https://github.com/watterm/llm-metadata-annotation/tree/pilot-simplified.

Figure 3a: Detailed results of the initial run of Step 2 using Gemini 2.5 Pro according to the prompting scheme in Supplemental Figure 1

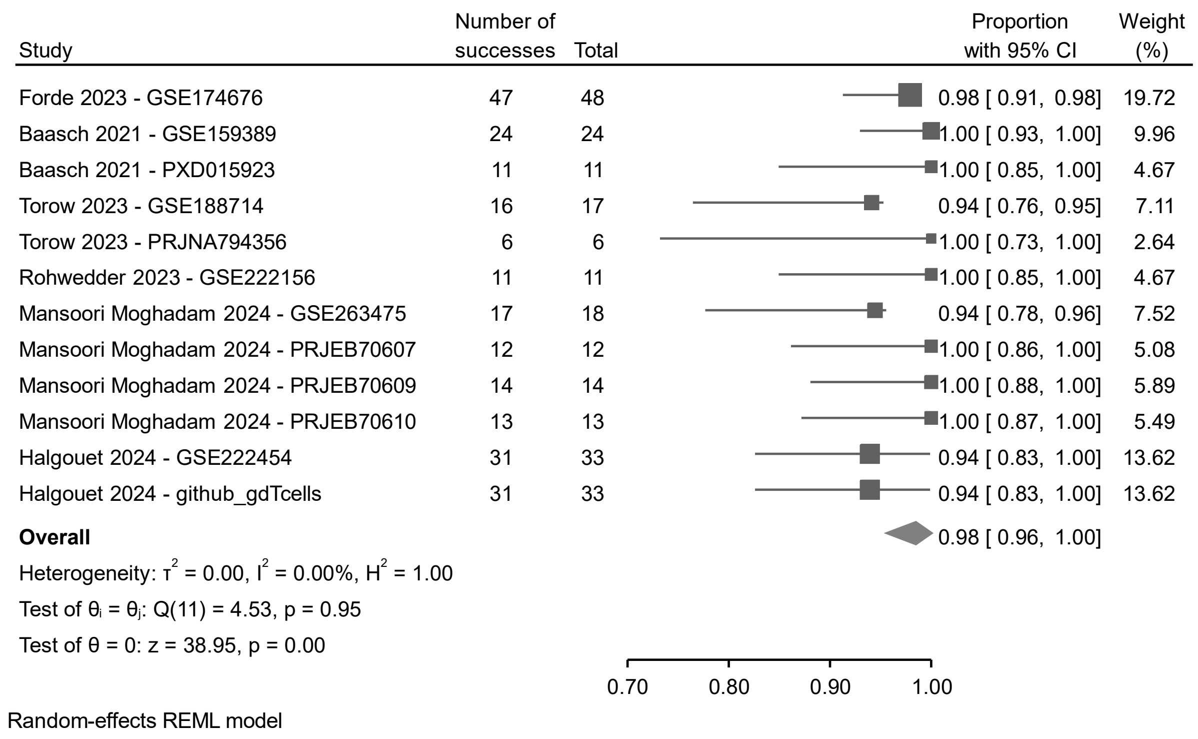

Figure 3b: Detailed results of the initial run of Step 2 using Gemini 2.5 Pro according to the new implementation of the approach as outlined at https://github.com/watterm/llm-metadata-annotation/tree/pilot-simplified

Study	Number of successes	Total	Proportion with 95% CI	Weight (%)
Forde 2023 - GSE174676	20	20	1.00 [0.92, 1.00]	10.44
Baasch 2021 - GSE159389	16	16	1.00 [0.90, 1.00]	8.98
Baasch 2021 - PXD015923	10	10	1.00 [0.83, 1.00]	6.36
Torow 2023 - GSE188714	16	18	0.89 [0.69, 1.00]	9.73
Torow 2023 - PRJNA794356	5	7	0.71 [0.32, 0.99]	4.82
Rohwedder 2023 - GSE222156	12	12	1.00 [0.86, 1.00]	7.30
Mansoori Moghadam 2024 - GSE263475	13	17	0.76 [0.53, 0.94]	9.36
Mansoori Moghadam 2024 - PRJEB70607	12	13	0.92 [0.70, 0.94]	7.74
Mansoori Moghadam 2024 - PRJEB70609	11	12	0.92 [0.68, 0.93]	7.30
Mansoori Moghadam 2024 - PRJEB70610	12	13	0.92 [0.70, 0.94]	7.74
Halgouet 2024 - GSE222454	33	34	0.97 [0.88, 0.98]	14.37
Halgouet 2024 - github_gdTcells	8	9	0.89 [0.58, 0.91]	5.87
**Overall**			0.95 [0.90, 0.99]	

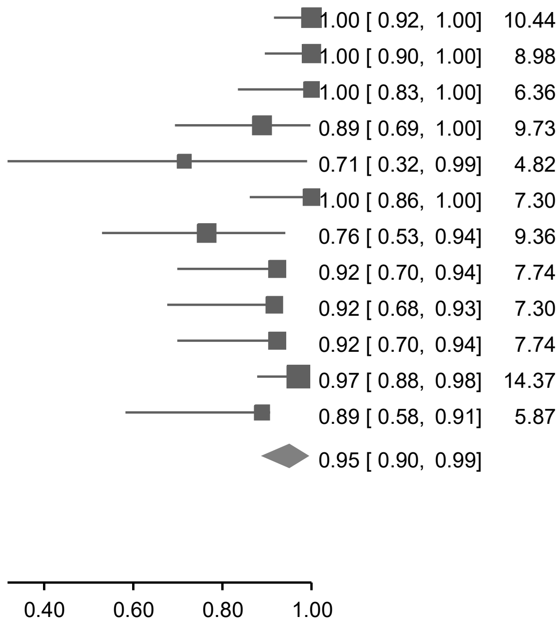

Heterogeneity: $\tau^2 = 0.02$, $I^2 = 26.55\%$, $H^2 = 1.36$

Test of $\theta_i = \theta_j$: Q(11) = 14.99, p = 0.18

Test of θ = 0: z = 26.47, p = 0.00

Random-effects REML model

Figure 3c: Calculation of the Jaccard Index between the initial run and the new implementation of the approach. Number of successes represent overlapping entities between the two runs.

Study	Number of successes	Total	Proportion with 95% CI	Weight (%)
Forde 2023 - GSE174676	20	48	0.42 [0.28, 0.56]	10.10
Baasch 2021 - GSE159389	15	25	0.60 [0.40, 0.79]	8.97
Baasch 2021 - PXD015923	9	12	0.75 [0.46, 0.96]	7.22
Torow 2023 - GSE188714	14	21	0.67 [0.45, 0.86]	8.60
Torow 2023 - PRJNA794356	6	7	0.86 [0.48, 0.88]	5.74
Rohwedder 2023 - GSE222156	10	13	0.77 [0.50, 0.96]	7.43
Mansoori Moghadam 2024 - GSE263475	14	21	0.67 [0.45, 0.86]	8.60
Mansoori Moghadam 2024 - PRJEB70607	9	16	0.56 [0.31, 0.80]	7.96
Mansoori Moghadam 2024 - PRJEB70609	9	17	0.53 [0.29, 0.76]	8.11
Mansoori Moghadam 2024 - PRJEB70610	9	17	0.53 [0.29, 0.76]	8.11
Halgouet 2024 - GSE222454	32	35	0.91 [0.79, 0.99]	9.61
Halgouet 2024 - github_gdTcells	8	34	0.24 [0.11, 0.39]	9.56
**Overall**			0.62 [0.49, 0.74]	

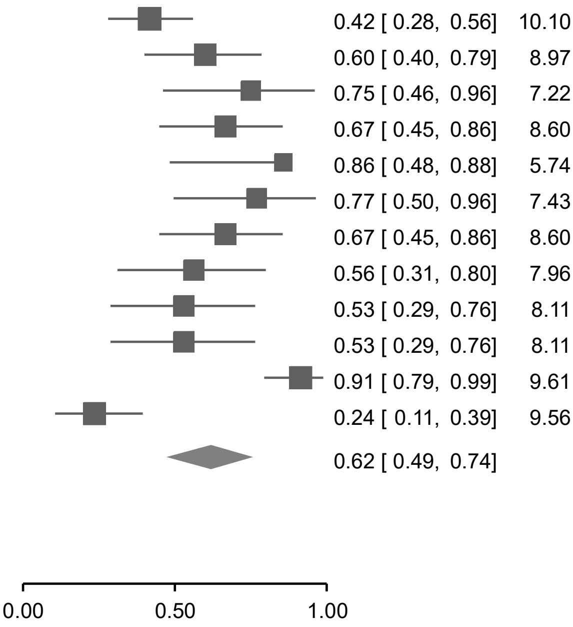

Heterogeneity: $\tau^2 = 0.13$, $I^2 = 73.96\%$, $H^2 = 3.84$

Test of $\theta_i = \theta_j$: Q(11) = 50.22, p = 0.00

Test of θ = 0: z = 12.74, p = 0.00

Random-effects REML model
